# EmoShiftNet: a shift-aware multi-task learning framework with fusion strategies for emotion recognition in multi-party conversations

**DOI:** 10.3389/frai.2025.1618698

**Published:** 2025-09-03

**Authors:** Hinduja Nirujan, Y. H. P. P. Priyadarshana

**Affiliations:** ^1^Informatics Institute of Technology, Colombo, Sri Lanka; ^2^Kyoto University of Advanced Science, Kyoto, Japan

**Keywords:** emotion recognition, emotion shift detection, deep learning, multimodal emotion recognition, speech emotion analysis, multi-party conversations

## Abstract

**Introduction:**

Emotion Recognition in Conversations (ERC) is vital for applications such as mental health monitoring, virtual assistants, and human–computer interaction. However, existing ERC models often neglect emotion shifts—transitions between emotional states across dialogue turns in multi-party conversations (MPCs). These shifts are subtle, context-dependent, and complicated by class imbalance in datasets such as the Multimodal EmotionLines Dataset (MELD).

**Methods:**

To address this, we propose EmoShiftNet, a shift-aware multi-task learning (MTL) framework that jointly performs emotion classification and emotion shift detection. The model integrates multimodal features, including contextualized text embeddings from BERT, acoustic features (Mel-Frequency Cepstral Coefficients, pitch, loudness), and temporal cues (pause duration, speaker overlap, utterance length). Emotion shift detection is incorporated as an auxiliary task via a composite loss function combining focal loss, binary cross-entropy, and triplet margin loss.

**Results:**

Evaluations on the MELD dataset demonstrate that EmoShiftNet achieves higher overall F1-scores than both traditional and graph-based ERC models. In addition, the framework improves the recognition of minority emotions under imbalanced conditions, confirming the effectiveness of incorporating shift supervision and multimodal fusion.

**Discussion:**

These findings highlight the importance of modeling emotional transitions in ERC. By leveraging multi-task learning with explicit shift detection, EmoShiftNet enhances contextual awareness and offers more robust performance for multi-party conversational emotion recognition.

## 1 Introduction

Emotion Recognition in Conversations (ERC) has become an essential component in human-computer interaction (HCI), sentiment analysis, mental health monitoring, and intelligent virtual assistants. Understanding human emotions is critical for enabling machines to engage in natural, empathetic, and context-aware communication (Hazarika et al., 2021). However, the dynamic nature of human emotions in conversations, particularly in multi-party settings, presents significant challenges for existing ERC models. One of the most overlooked aspects in current ERC research is the detection of emotion shifts, which refer to the transitions between emotional states during a conversation. Traditional ERC models often assume that emotions remain static within a given conversational turn. However, human emotions are fluid and context-dependent, influenced by previous utterances, speaker interactions, and external factors. Emotion shifts occur frequently in natural dialogues, yet most ERC models fail to account for these transitions (Majumder et al., 2019). This limitation reduces the accuracy and adaptability of ERC systems as they struggle to capture subtle variations and abrupt emotional changes.

In multi-party conversations (MPCs), where multiple speakers contribute to an evolving emotional landscape, detecting when and how emotions shift is crucial for improving emotion recognition performance (Gao et al., 2022; Hazarika et al., 2018a). Recent advancements in ERC have focused on modeling inter-speaker dependencies, leveraging multimodal signals, and incorporating contextual memory mechanisms. For instance, DialogueGCN (Ghosal et al., 2019) employs graph convolutional networks to capture speaker-specific and context-aware features, while DialogueCRN (Hu et al., 2021) introduces a recurrent attention mechanism to model dynamic emotional states. Transformer-based methods like DialogueEIN (Li et al., 2023) exploit self-attention and speaker embeddings to improve context modeling. Despite these innovations, most models still treat emotion shifts implicitly, lacking explicit mechanisms to identify transitions between emotional states.

A few recent studies have attempted to incorporate emotion shift detection via multi-task learning (MTL) (Wang et al., 2023; Gao et al., 2022). However, these are largely limited to speaker-level or sentiment-based shift definitions, and do not exploit acoustic or temporal features. Additionally, they lack control ablations to verify the effectiveness of shift supervision in improving ERC. To address this challenge, this study introduces EmoShiftNet, a shift-aware, multi-task learning framework that jointly models emotion recognition and binary global emotion shift detection. Unlike prior work, EmoShiftNet incorporates multimodal fusion of contextualized text, acoustic prosody, and explicit temporal dynamics such as pause durations, speaker overlap to model emotional transitions. The model is evaluated on the Multimodal EmotionLines Dataset (MELD), a benchmark for MPCs with rich dialogue and emotional annotations. Although MELD and similar datasets provide utterance-level emotion labels, most existing ERC models do not explicitly model the temporal dynamics of emotion transitions between adjacent utterances. Instead, they treat each utterance in isolation or rely on static, global context representations. Our approach, in contrast, explicitly incorporates emotion shift modeling between utterances as an auxiliary task to guide the main classification objective. Experiments show that EmoShiftNet achieves state-of-the-art performance in both overall and minority emotion classification, and a control ablation with shuffled shift labels confirms the contribution of meaningful shift supervision to emotion recognition performance.

The key contributions of this work are:

- A novel shift-aware ERC framework that integrates global emotion shift detection using multi-task learning, addressing a critical limitation of traditional ERC models.
- A multimodal fusion strategy that combines textual, acoustic, and temporal features, capturing dynamic emotional transitions across speakers and dialogue turns.
- Extensive evaluation and control ablations, showing that EmoShiftNet outperforms strong baselines in both accuracy and minority emotion detection, especially under class imbalance.

The rest of the paper is organized as follows: section 2 reviews related work in emotion recognition, multi-task learning, and emotion shift detection. Section 3 details the dataset, feature processing techniques, and experimental design. Section 4 presents the results and empirical findings, followed by section 5, which discusses key insights and future directions. Finally, section 6 concludes the paper with a summary of contributions and impact.

## 2 Related work

### 2.1 Emotion recognition in conversations

ERC has gained increasing attention with advancements in deep learning and the availability of large-scale conversational datasets. Traditional ERC models focus on capturing static contextual information, such as speaker embeddings and previous utterances, to infer emotional states (Cai et al., 2024). Early approaches, such as DialogueRNN (Majumder et al., 2019), employed recurrent neural networks to track the emotional state of each participant, modeling the evolving context over multiple turns. More recent work, including COSMIC (Ghosal et al., 2020), introduced external knowledge to improve the understanding of speaker interactions, while DAG-ERC (Shen et al., 2021) leveraged directed acyclic graph structures to capture long-distance dependencies in conversations. Other studies have focused on enhancing context modeling by incorporating reasoning and interaction networks. For instance, hierarchical multimodal fusion has been shown to outperform simple concatenation in multimodal sentiment analysis (Majumder et al., 2018). DialogueCRN (Hu et al., 2021) introduced perceptual reasoning based on emotion theory to improve emotion classification. CoMPM (Lee and Lee, 2022) proposed a model that explicitly extracts contextual features and integrates them into pre-trained language models, significantly improving ERC performance. However, these methods focus on modeling speaker states and conversation history but omit the dynamic progression of emotions over time.

Recently, researchers have started addressing the challenge of dynamic emotional transitions in ERC by modeling emotion propagation and inter-speaker dependencies. EmotionFlow (Song et al., 2022) introduced a Conditional Random Field (CRF) layer to capture emotion transfer patterns across utterances. HCL-ERC (Yang et al., 2022) applied curriculum learning to model emotion shifts, progressively introducing training examples based on the frequency of detected shifts. DialogueEIN (Liu et al., 2022) proposed an emotion interaction network that jointly models intra-speaker, inter-speaker, and global–local emotional dependencies to improve ERC in multi-party settings. These studies highlight the growing recognition of emotion dynamics in ERC, underscoring the need for emotion shift detection as a complementary task to traditional emotion classification.

Earlier multimodal work demonstrated the importance of capturing interactions between modalities (Zadeh et al., 2016). Additionally, contrastive representation learning has gained traction in ERC. Joyful (Hu et al., 2023) combined joint modality fusion with graph contrastive learning to align multimodal representations, while Emp (Wang et al., 2023) guided modality fusion using auxiliary emotion predictions and contrastive loss for personality trait recognition. These works affirm the utility of auxiliary supervision and fusion learning, though neither explicitly models emotion shift phenomena — a core focus of this work.

### 2.2 Emotion shift detection in MPCs

Emotion shifts refer to the transition from one emotional state to another within a conversation, often influenced by context, speaker interactions, and the dialogue flow. Traditional ERC models treat emotion recognition as a static classification problem, assigning a fixed label to each utterance. However, real-world conversations involve frequent emotional changes, where a speaker may shift from neutral to frustration, sadness to joy, or anger to calmness, depending on the ongoing interaction (Chen et al., 2022). These transitions play a crucial role in understanding conversational intent and constructing adaptive AI systems that can respond appropriately to evolving emotions.

One of the major challenges in emotion shift detection is the prevalence of neutral emotions, which dominate most datasets, making it difficult to detect low frequency but significant shifts. Speaker interactions complicate the detection of emotion shifts, as conversational flow and emotion transmission affect how emotions are expressed and received in dialogue. Various approaches have been proposed to model emotion shifts dynamically. Early methods relied on rule-based and statistical models, using handcrafted features to identify shifts in sentiment polarity or vocal tone (Wang et al., 2025). Hidden Markov Models (HMMs) were later applied to sequentially model emotion transitions, allowing for probabilistic estimation of shift occurrences (Le and Provost, 2013). However, these methods were limited in capturing speaker dependencies and long-range context. More recently, deep learning models have evolved with context-aware architectures to detect emotion shifts. Zhao et al. (2018) incorporated attention-based BiLSTM-RNNs, enhancing temporal modeling for emotion progression. Transformer-based models now represent the state-of-the-art in ERC, with specialized adaptations focusing on emotion dynamics and speaker-aware modeling (Wang and Mine, 2023).

### 2.3 MTL in emotion recognition

MTL is an effective machine learning paradigm where a model simultaneously learns multiple related tasks, utilizing shared information to improve generalization. In ERC, MTL has been successfully employed to integrate auxiliary tasks such as speaker identification, sentiment classification, and emotion shift detection to enhance model robustness. One of the earliest applications of MTL in ERC was the study by Li et al. (2020), where speaker identification was used as an auxiliary task to strengthen context modeling. Subsequently, Gao et al. (2022) proposed ERC-ESD, using speaker-level emotion shift detection within an MTL setup. While their results showed performance gains, their modeling was limited to intra-speaker shifts, overlooking inter-speaker dynamics common in multi-party conversations. In a more recent study, Wang et al. (2023) employed MTL with both sentiment classification and emotion shift detection as auxiliary tasks. Their approach modeled emotion shifts based on sentiment polarity differences between consecutive utterances. However, both of their framework relied solely on textual features and did not evaluate the specific contribution of each auxiliary task through ablation.

The performance of an MTL model depends on the task relevance. When the auxiliary task has meaningful relationships with the primary task, the model performance will be enhanced. For example, Chauhan et al. (2020) explored MTL in sarcasm detection, leveraging emotion recognition and sentiment classification as auxiliary tasks. Similarly, sentiment classification has been shown to enhance emotion recognition, as coarse-grained sentiment labels provide additional context for fine-grained emotion categories. Within the scope of ERC, emotion shifts, sentiment polarity, and emotion classification share a strong conceptual relationship, as all three involve the temporal progression of emotional states. This study builds on prior work by explicitly integrating emotion shift detection into an MTL framework. It hypothesizes that shift detection serves as an essential auxiliary task that reinforces emotion recognition by providing additional learning signals.

### 2.4 Research gap

Despite recent advancements in ERC, most existing models treat emotional states as static, ignoring the dynamic transitions that naturally occur in conversations. While some work has explored emotion propagation and context modeling, emotion shift detection remains under-addressed as a dedicated learning objective, particularly in multi-party settings where inter-speaker interactions drive emotional changes. Moreover, although multi-task learning has shown promise in ERC, limited research has combined it with explicit shift detection and multimodal fusion strategies. Existing studies often overlook how these components interact, especially in imbalanced datasets, where recognizing low-frequency emotional shifts is critical. To address these limitations, this study proposes a shift-aware, multi-task framework that integrates emotion classification and shift detection, leveraging textual, acoustic, and temporal fusion for enhanced contextual modeling. The work is guided by the following research questions:

*RQ1*: What are the limitations of current Emotion Recognition in Conversation (ERC) models in multi-party settings?

*RQ2*: How does the proposed framework compare with existing ERC models in terms of performance?

*RQ3*: What techniques are most effective for detecting both subtle and abrupt emotional shifts in conversations?

*RQ4*: How can incorporating emotion shift detection improve ERC performance?

These questions form the foundation of the proposed research, aiming to advance the field by explicitly modeling emotion shifts, addressing class imbalance, and improving multimodal emotion understanding.

## 3 Methods

### 3.1 Dataset selection—MELD dataset

This study utilizes the MELD, a well-established benchmark for emotion recognition in MPCs. Introduced by Poria et al., MELD is derived from the TV series Friends and consists of over 13,000 utterances across 1,400 + dialogues, each annotated with one of seven emotion labels: anger, disgust, fear, joy, neutral, sadness, and surprise. The dataset includes textual transcripts, audio recordings, and visual cues, along with metadata for speaker and dialogue context, enabling multimodal learning for emotion modeling. As outlined in Table 1, MELD offers significant diversity in emotional expression and conversational structure. Each dialogue contains an average of 3.3 distinct emotions, making it highly suitable for investigating emotion shifts. In total, MELD contains over 5,400 annotated global-level emotion transitions, where the emotional state changes between consecutive utterances within a dialogue, regardless of the speaker.

**Table 1:** MELD dataset statistics and structure.

Aspect	Details
Total dialogues	1,433
Total utterances	13,708
Number of speakers	260 unique speakers
Average utterance length	~8 s
Emotion labels	7 classes: anger, disgust, fear, joy, neutral, sadness, surprise
Avg. distinct emotions per dialogue	3.3
Total labeled emotion shifts	5,404
Mean shifts per dialogue	3.55

The table summarizes the number of dialogues, utterances, speakers, and labeled global emotion shifts, illustrating the dataset’s richness and suitability for multi-party emotion recognition.

To better understand the distribution of emotion shifts, an exploratory analysis was conducted on the MELD training set. This analysis revealed that emotion transitions frequently originate from the neutral class, with neutral → joy being among the most common. To analyze the frequency of emotion shifts relative to the number of utterances, a shift density measure was calculated for the top 15 speakers.


$$\text{Shift density ratio}=\frac{\text{number of shifts}\;\mathrm{by}\;\text{the speaker}}{\text{total number of utterances of the speaker}}$$

**Figure 1:**
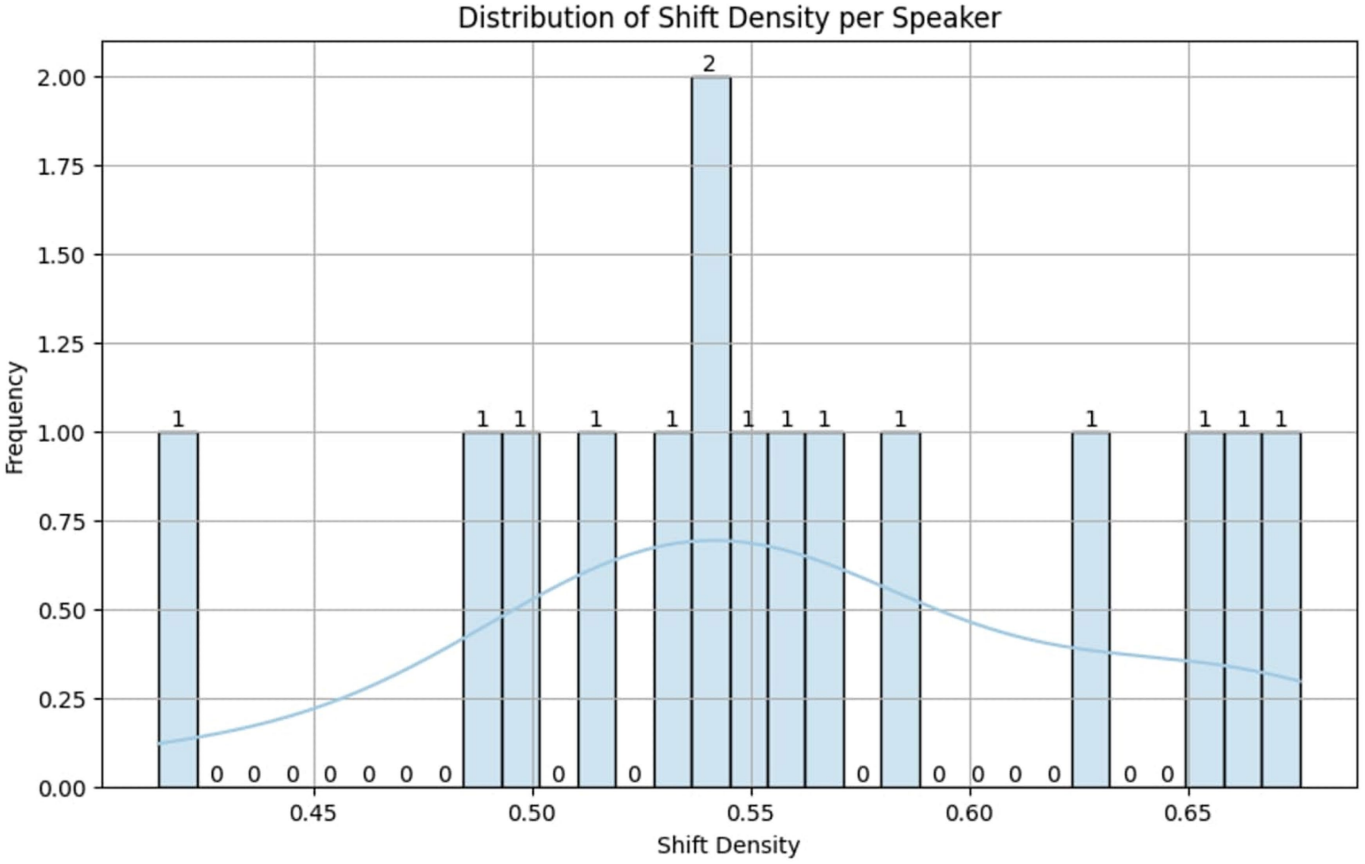
Distribution of emotion shift density per speaker in the MELD dataset. The histogram illustrates how frequently speakers experience emotional transitions, confirming high emotional variability across conversations and supporting the need for shift-aware emotion recognition.

This ratio helps to identify speakers who experience more frequent emotional transitions, and a histogram of shift densities provided in Figure 1 confirmed that emotional variability is high across dialogues, supporting the need for shift-aware learning models. These statistics emphasize that MELD not only offers rich emotional diversity but also provides a realistic conversational structure for modeling emotional transitions over time—making it an ideal foundation for the proposed EmoShiftNet framework.

**Figure 2:**
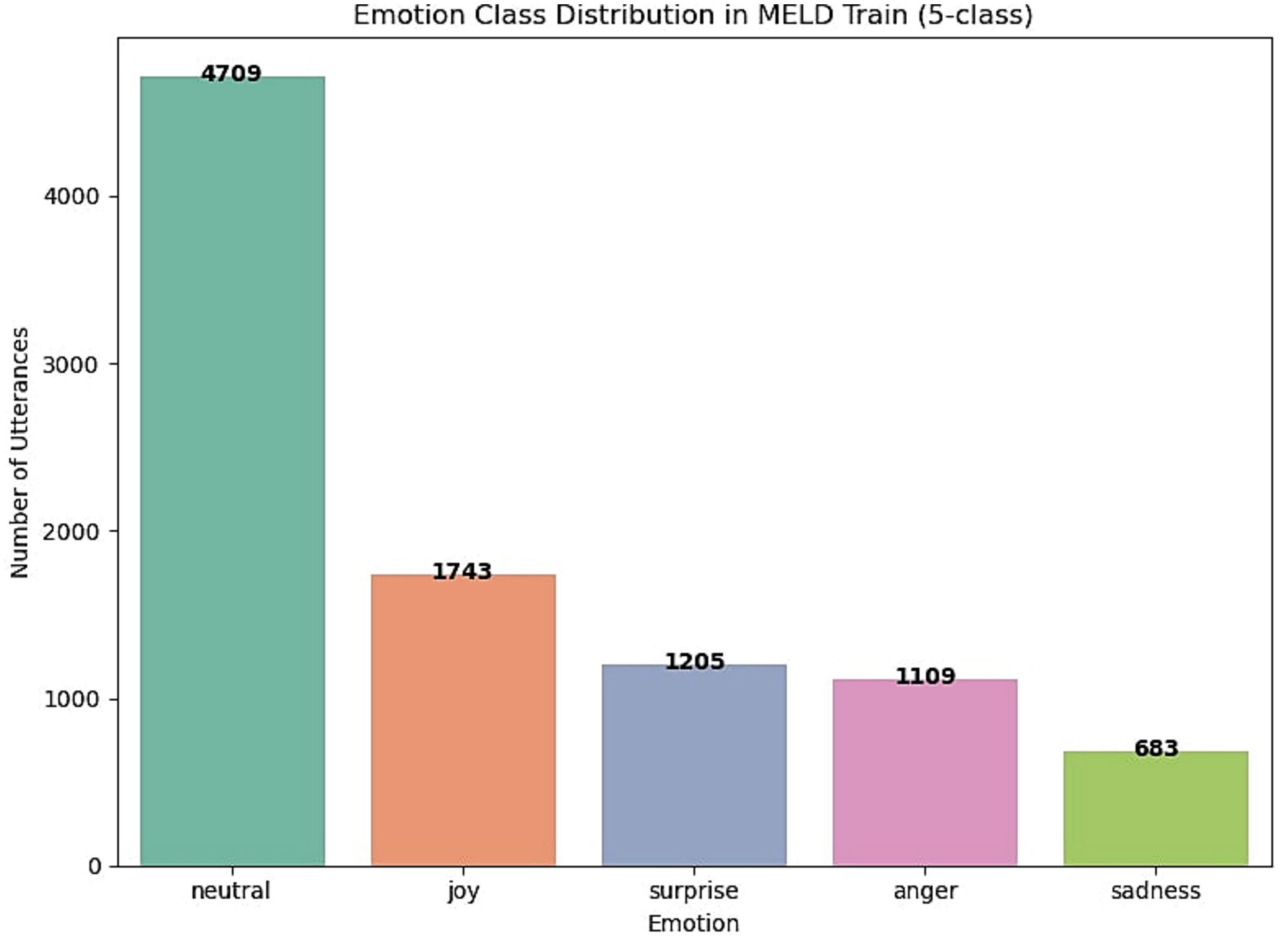
Emotion label distribution in the MELD training set (5-class subset). The neutral class is the majority class, while sadness, anger, and surprise appear far less frequently, contributing to the dataset’s inherent class imbalance.

Along with emotion shift variability, MELD exhibits a pronounced class imbalance, as illustrated in Figure 2. The neutral class overwhelmingly dominates the label distribution, accounting for over 4,709 utterances in the training set. In contrast, minority emotions such as sadness (683 samples), anger (1,109), and surprise (1,205) are significantly underrepresented. This imbalance poses a challenge for effective emotion classification, particularly for detecting minority-class utterances under real-world conditions. Addressing this skewed distribution is therefore a key focus of the proposed EmoShiftNet model.

In addition to MELD, the Interactive Emotional Dyadic Motion Capture (IEMOCAP) dataset was used to evaluate the generalizability of EmoShiftNet. IEMOCAP contains dyadic conversations annotated for categorical emotions. To ensure consistency, we selected five overlapping emotion categories: neutral, joy, anger, sadness, and surprise. The same feature extraction and training pipeline was applied, allowing for a comparative analysis under similar experimental settings.

### 3.2 Preprocessing and feature engineering

To ensure high-quality input representations, a multi-stage preprocessing pipeline was applied to extract meaningful linguistic, acoustic, and temporal features. This pipeline aimed to reduce noise, standardize input formats, and enhance the model’s ability to detect subtle emotion shifts by leveraging both text and speech modalities.

#### 3.2.1 Text preprocessing

Textual data was preprocessed using tokenization, normalization, and embedding extraction to ensure consistent and context-aware representations. A BERT-based tokenizer segmented text into sub word units, efficiently handling out-of-vocabulary words. The processed tokens were then passed through pre-trained transformer embeddings, which were fine-tuned on conversational datasets to capture emotion-specific linguistic variations.

#### 3.2.2 Audio preprocessing

The audio preprocessing pipeline was designed to ensure consistent and high-quality feature extraction from conversational audio, which often includes overlapping speech, ambient noise, and inter-speaker variability. To address these challenges, several sequential processing steps were applied. First, noise reduction techniques such as spectral subtraction and adaptive filtering were used to enhance the clarity of speech while minimizing the impact of background noise. This step was critical for improving the signal-to-noise ratio, especially in natural multi-party conversations where extraneous sound is common.

Following noise reduction, voice activity detection (VAD) was employed to isolate segments containing actual speech. The audio was divided into small frames, each analyzed by a VAD model to determine whether it contained speech. Only the frames identified as speech were retained and recombined, effectively removing silent and non-speech regions. Finally, all audio samples were resampled to a uniform 16 kHz sampling rate, which standardizes the temporal resolution across the dataset and reduces variability in downstream acoustic feature extraction. This preprocessing pipeline ensured that the extracted features were clean, consistent, and suitable for robust modeling in emotion recognition tasks.

#### 3.2.3 Feature engineering

To jointly model emotion classification and emotion shift detection, a MTL framework was adopted, where emotion recognition was treated as the primary task and shift detection served as an auxiliary task. The model leveraged a multimodal feature integration strategy, combining linguistic, acoustic, and temporal features to enhance performance across both tasks. Linguistic inputs were extracted using 768-dimensional BERT-based embeddings, which provided context-aware representations of each utterance. These embeddings were fine-tuned on conversational data to better capture emotion-dependent language patterns and improve the model’s sensitivity to subtle textual cues.

In addition to textual signals, acoustic features were incorporated, including 13-dimensional Mel-Frequency Cepstral Coefficients (MFCCs) to represent spectral properties of speech, along with pitch and loudness to capture variations in intonation and vocal intensity. To further model conversational dynamics, temporal features such as utterance duration, pause duration, and overlapping ratio were used to quantify speaking behavior and interaction patterns between participants. Following initial benchmarking experiments, an attention based fusion strategy was applied to integrate all three feature types at the input level. This method enabled the model to learn richer feature interactions and proved particularly effective in addressing class imbalance, improving the detection of emotion shifts in underrepresented emotional categories.

### 3.3 MTL-based emotion recognition and emotion shift detection

**Figure 3:**
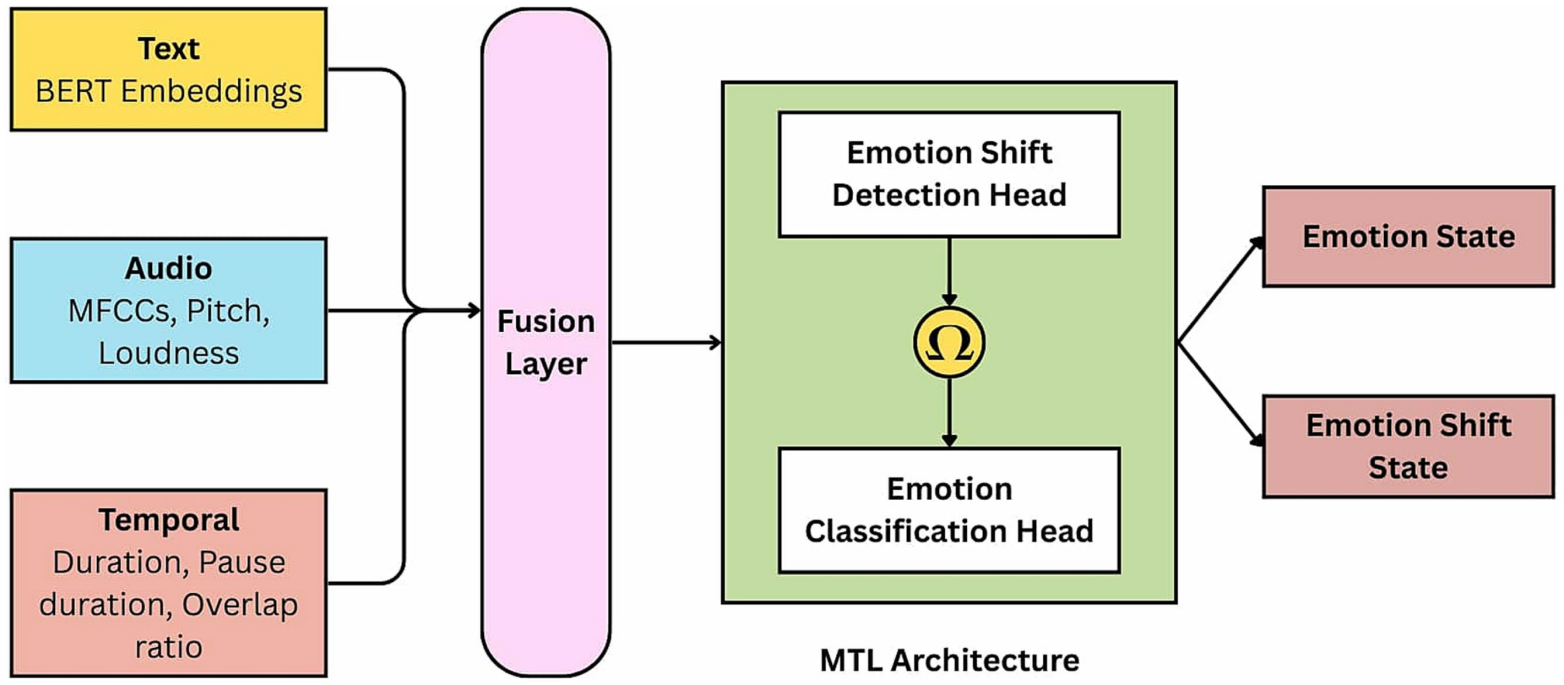
Overall architecture of the proposed EmoShiftNet framework. The model consists of three core components: feature extraction modules, a multimodal fusion layer, and task-specific learning heads for both emotion classification and shift detection.

EmoShiftNet adopts a MTL framework that integrates emotion recognition and emotion shift detection to improve performance in MPCs. By jointly learning both tasks, the model captures shared representations that enhance its ability to detect dynamic emotional transitions while mitigating the effects of class imbalance. The overall architecture of the EmoShiftNet is shown in Figure 3. It comprises three main components: feature extraction, multimodal fusion, and task-specific learning heads.

#### 3.3.1 Emotion shift annotation

Emotion shift detection was formulated as a binary classification task indicating whether an utterance marks a change in emotional state from the previous utterance within the same speaker turn. Given an utterance 𝑢_𝑡_ with emotion label 𝑒_𝑡_, and the immediately preceding utterance 𝑢_𝑡−1_ with emotion 𝑒_𝑡−1_, a shift label 𝑠_𝑡_ was computed as:


$$s_{t}=f(x)=\{\begin{array}{c} 1,\text{if}\;e_{t}\neq e_{t-1}\;\text{and same dialogue}\\ 0,\text{otherwise} \end{array}$$

This logic was applied across all dialogues, ensuring that emotion shifts were annotated in a context-aware manner. Similar approaches have been used in prior work (Ghosal et al., 2020), and our implementation extends these strategies to a multi-party conversational setting with multimodal input.

#### 3.3.2 Multimodal fusion strategy

Given the challenges posed by imbalanced emotion distributions in conversational datasets, several fusion techniques were explored to effectively integrate textual, acoustic, and temporal features. Fusion strategies play a crucial role in aligning and leveraging complementary information from multiple modalities, particularly in scenarios where certain emotional categories are underrepresented (Hazarika et al., 2018a; Poria et al., 2019).

**Figure 4:**
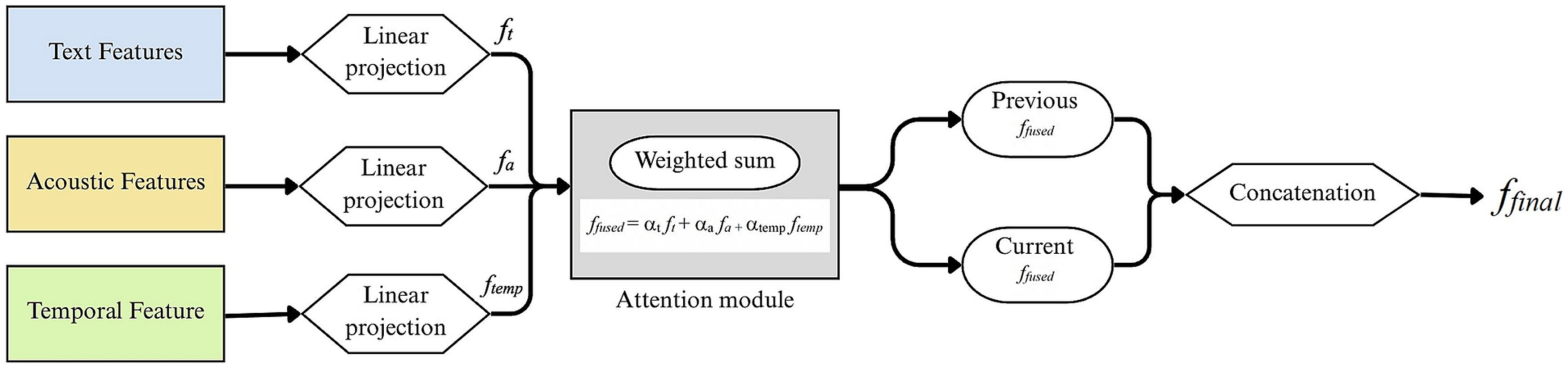
Overall architecture of the proposed attention-based multimodal fusion framework.

The proposed framework incorporates a dedicated attention-based fusion strategy to dynamically integrate modality-specific information. The method first projects each modality into a shared latent space and then applies learnable attention weights to determine the relative importance of each modality for every utterance. As illustrated in Figure 4, the fusion module processes each utterance in a pairwise context (i.e., previous and current utterances). Specifically, each modality the from BERT-based textual features, acoustic features and temporal features is first passed through a separate linear projection layer to map it into a common dimensionality space R^d^, where *d* = 128.

For a given utterance, the attention weights over modalities are computed using.

*f_fused_* = α_t_ *f_t_* + α_a_ *f*_*a* +_ α_temp_ *f_temp_* where, $\alpha_{i}=\frac{\exp(\omega_{i}^{T}f_{i})}{\sum_{j}\exp(\omega_{j}^{T}f_{j}).}$

Here the variables 𝑓_𝑡_, 𝑓_𝑎_, and 𝑓_temp_ represent the linearly projected features from the textual, acoustic, and temporal modalities, respectively. Each modality-specific feature vector is first processed through a linear projection layer to ensure dimensional alignment. In the attention module, each modality *𝑖 ∈ {t, 𝑎, temp}* is assigned a learnable weight vector *ω_i_* which governs the importance of that modality in the final fusion. The attention weights $\alpha_{i}$ are computed for each modality based on *ω_i_*, enabling the model to dynamically prioritize modalities depending on their contextual relevance to the current utterance.

The fusion process is then applied independently to the previous (f_prev_) and current (f_curr_) utterance feature vectors, and the final multimodal input is obtained by concatenating these two fused representations:


$$f_{\text{final}}=\text{Concat}\;(f_{\text{prev}},f_{\text{curr}}\;)$$

This design enables context-aware fusion that adapts the contribution of each modality based on the content of the utterance. The attention mechanism captures the variability, enhancing both emotion recognition and emotion shift detection performance. Moreover, this late fusion architecture avoids the pitfalls of early fusion, such as feature dominance and misalignment across modalities (Zhao et al., 2013).

#### 3.3.3 Baseline model: MTL architecture

The baseline model, EmoShiftNet, is a MTL architecture built upon a feedforward neural network (FFNN). It jointly optimizes two related tasks: emotion recognition (primary) and emotion shift detection (auxiliary), enabling shared learning from multimodal input features.

##### 3.3.3.1 Shared feature encoder

After extracting and fusing text, acoustic, and temporal features from both the current and previous utterances, the fused 256-dimensional vector is passed through a shared encoder comprising multiple fully connected layers. Specifically, the shared encoder contains two linear layers interleaved with ReLU activations, Layer Normalization, and Dropout, producing a 512-dimensional latent representation. This module captures high-level cross-modal interactions crucial for both emotion recognition and shift detection.

##### 3.3.3.2 Task-specific heads

- Emotion Classification Head:
  A fully connected output layer maps the shared representation to logits over the predefined set of emotion classes (e.g., joy, anger, sadness). The model predicts the emotion class by applying a SoftMax over these logits.
- Shift Detection Head:
  A separate output layer projects the same shared representation to a single scalar, which is passed through a sigmoid activation to estimate the likelihood of an emotion shift as binary classification.

The model is trained using a composite loss function that combines

- Label-Smoothed Focal Loss for emotion classification (𝐿_emotion_),
- Binary Cross-Entropy Loss for shift detection (𝐿_shift_),
- Triplet Margin Loss to encourage intra-class compactness and inter-class separability in the embedding space (𝐿_triplet_).

The total loss is defined as:


$$L_{\text{total}}=L_{\text{emotion}}+\lambda .L_{\text{shift}}+\gamma \cdot L_{\text{triplet}}$$

where *λ* and *γ* are hyperparameters that balance the contributions of each component.

**Figure 5:**
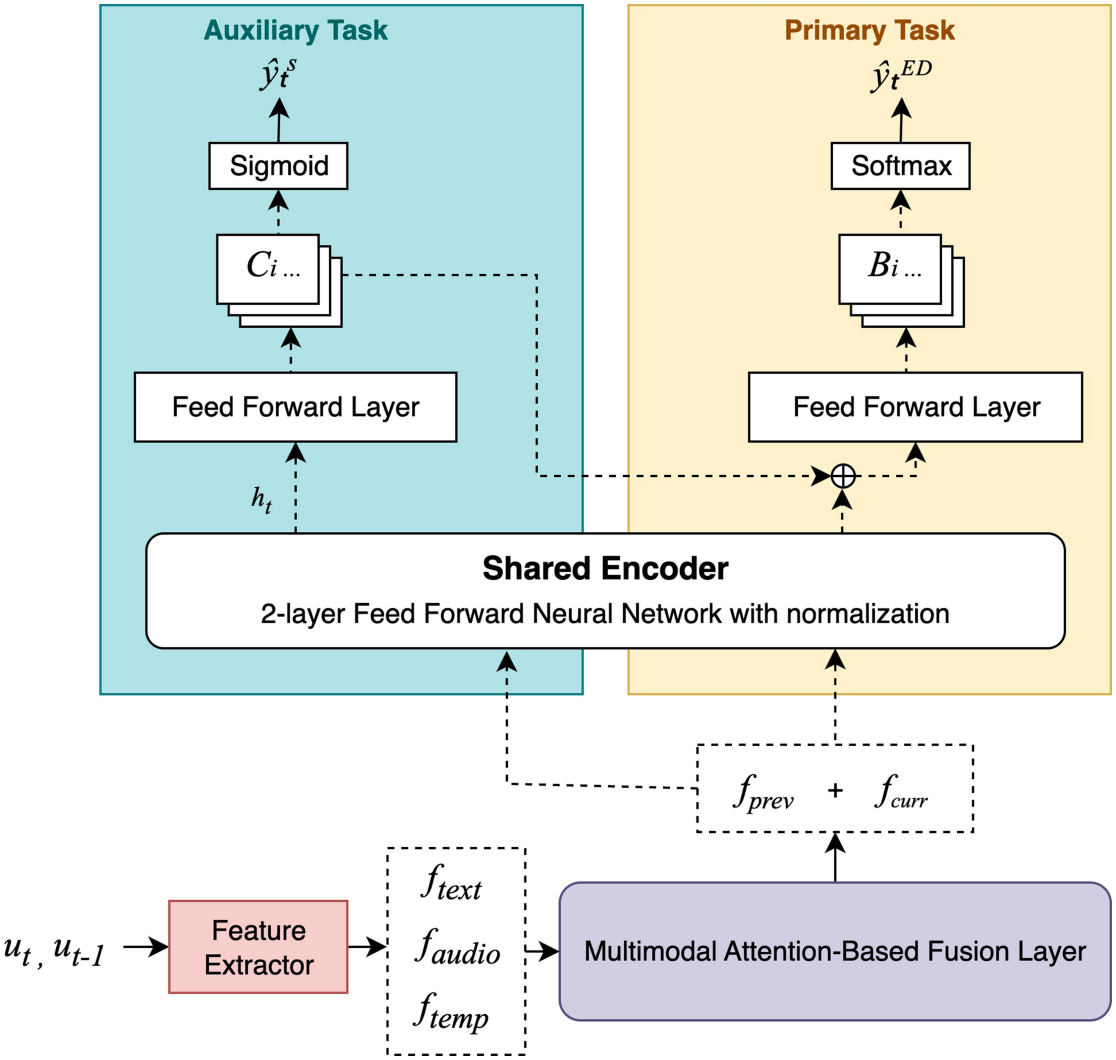
Overview of EmoShiftNet architecture. The model uses a shared encoder to jointly perform emotion classification and emotion shift detection.

Label smoothing mitigates model overconfidence and improves generalization on minority emotion classes (Szegedy et al., 2015), while triplet loss encourages clustering of emotion-specific embeddings in latent space (Schroff et al., 2015). The triplet loss is computed here by mining anchor-positive–negative samples within the same batch. Positive samples are utterances sharing the same emotion label as the anchor, while negatives belong to different emotion classes. The model is optimized using the Adam optimizer with a constant learning rate. The architecture and flow of this model are illustrated in Figure 5, highlighting the fusion mechanism, shared encoder, and dual-head design.

### 3.4 Experimental setup

To evaluate the effectiveness of EmoShiftNet in recognizing emotions and detecting emotion shifts in multi-party conversations, a series of controlled experiments were conducted. This section outlines the evaluation framework that systematically describes datasets, implementation details, baseline comparisons, model tuning strategies, and evaluation settings.

#### 3.4.1 Datasets and implementation details

The experiments are conducted on the MELD and IEMOCAP datasets in filtered (5-class) variant. IEMOCAP is used to validate generalization. All the core logic was implemented in Python 3.8, using PyTorch, scikit-learn, and Google Cloud Storage APIs. Feature analysis and visualization were carried out using Pandas, Matplotlib, and Seaborn. Training and evaluation were conducted on a Google Cloud Platform (GCP) virtual machine (n1-standard-4, 4 vCPUs, 15 GB RAM).

#### 3.4.2 Benchmark evaluation

Comparative experiments were conducted using a filtered version of the MELD dataset containing the five most frequent emotion classes to ensure consistency across all models. The baseline models FFNN and RNN, graph-based architectures like DialogueGCN and DialogueCRN, and the proposed EmoShiftNet were all trained and evaluated using the same filtered data and feature set. To further explore the impact of class imbalance, an additional experiment was conducted on the MELD dataset using the EmoShiftNet model with SMOTE-based oversampling. This allowed us to isolate the effect of oversampling on minority emotion recognition without confounding model architecture differences.

#### 3.4.3 Training EmoShiftNet with multi-task learning

##### 3.4.3.1 Loss formulation and components

The second phase focuses on fine-tuning EmoShiftNet using multi-task learning. The model is trained using a composite loss function comprising three components: Label-Smoothed Focal Loss for emotion classification, Binary Cross-Entropy Loss for emotion shift detection, and Triplet Margin Loss to encourage intra-class compactness and inter-class separability in the shared embedding space. The total loss formulation is described in section 3.3.2.

To compute the triplet loss, we adopted random in-batch mining, where each anchor sample selects a positive counterpart sharing the same emotion label and a negative sample from a different class. Triplets were formed only when valid candidates existed within the current training batch, ensuring efficient computation and training stability. No inter-batch mining or hard/semi-hard mining was employed, as our goal was to maintain a balance between representation compactness and computational overhead under class-imbalanced conditions.

##### 3.4.3.2 Hyperparameter settings and sensitivity analysis of loss weights

Multiple values of 𝜆 and *γ* were empirically evaluated, and optimal settings were selected based on validation performance. A summary of all training hyperparameters is provided in Table 2. To analyze the effect of the multi-task loss components, we conduct a sensitivity analysis on the loss weights *λ* and γ. Table 3 summarizes the results, showing that the performance of EmoShiftNet is highly sensitive to the selection of these hyperparameters. Notably, the combination λ = 0.4 and γ = 0.1 achieves the highest test F1-score.

**Table 2:** Key hyperparameters used in EmoShiftNet training.

Hyperparameter	Values explored	Final selected value
Learning rate	1e-3, 5e-4, 1e-4, 5e-5	1e-4
Lambda (λ)	0.0, 0.2, 0.4, 0.6, 0.8, 1.0	0.4
Gamma (γ)	0.0, 0.05, 0.1, 0.2	0.1
Label smoothing factor	0.0, 0.1, 0.2	0.1
Epochs	10, 30, 50, 100	50

This includes learning rate, batch size, λ and γ loss weights, optimizer settings, and other training parameters used for model tuning.

**Table 3:** Sensitivity analysis of λ and γ on the MELD dataset using EmoShiftNet.

*λ* (Lambda)	γ (Gamma)	Average F1-score
0.0	0.0	0.3376
0.0	0.05	0.5085
0.2	0.1	0.5965
**0.4**	**0.1**	**0.6075**
0.4	0.2	0.5937
0.6	0.1	0.5913
0.8	0.1	0.5843
1.0	0.1	0.5473
1.0	0.2	0.2853

The table reports the average F1-score under different loss weight settings. Bold font denotes the best performances.

#### 3.4.4 Input modalities and contextual modeling

To assess the impact of different feature types and contextual information on model performance, multiple input configurations were designed and evaluated. These configurations varied based on the inclusion of textual, acoustic, and temporal features, as well as the use of previous utterance context. The goal was to identify which modalities and context combinations contribute most effectively to emotion recognition and emotion shift detection. Textual features were extracted using BERT-based embeddings, capturing contextual language information. Acoustic features included MFCCs, pitch, and loudness, providing prosodic and spectral cues. Temporal features such as utterance duration, pause duration, and overlapping ratio were added to model conversational pacing and speaker interaction dynamics. Two context modes were evaluated:

- Current utterance only
- Previous + current utterance combined

A summary of the input feature configurations used across experiments is shown in Table 4.

**Table 4:** Overview of input feature configurations used across experiments.

Configuration	Text	Audio	Temporal	Previous utterance
Text only	✓	╳	╳	╳
Text (with previous)	✓	╳	╳	
Text + Audio	✓	✓	╳	╳
Text + Audio (with previous)	✓	✓	╳	✓
Text + Temporal	✓	╳	✓	╳
Text + Temporal (with previous)	✓	╳	✓	✓
Text + Audio + Temporal	✓	✓	✓	╳
Full fusion	✓	✓	✓	✓

This includes combinations of text, audio, temporal features, and the presence or absence of previous utterance context.

#### 3.4.5 Fusion strategies

To investigate the impact of modality integration on multimodal emotion recognition, four fusion strategies were implemented and compared using the same filtered MELD dataset and model architecture:

Early Fusion: Simple concatenation of text, audio, and temporal features before feeding into the model.

- Weighted Fusion: Learnable scalar weights assigned to each modality to compute a weighted sum of modality projections.
- Attention-based Fusion: Modality-specific projections combined using a soft attention mechanism that dynamically highlights salient features.
- Transformer-based Fusion: Cross-modal interactions modeled using a lightweight transformer encoder to capture higher-order dependencies.
- All fusion modules were integrated with the same EmoShiftNet architecture to ensure fair comparison.

#### 3.4.6 Evaluation metrics and validation strategy

Model performance was evaluated using accuracy, F1-score, and confusion matrices for the multiclass emotion classification task. For the binary emotion shift detection task, accuracy was used as the primary metric. Each experiment was run with multiple random seeds to ensure robustness, and performance metrics were averaged across runs.

## 4 Results

This section presents the results of EmoShiftNet, in addressing emotion recognition and emotion shift detection in multi-party conversations. Results are organized around key experimental dimensions including model comparison, fusion strategies, loss function configurations, and ablation studies.

### 4.1 Benchmark performance across datasets

Table 5 provides a comparative overview of average F1-scores achieved by various model architectures across both the MELD and IEMOCAP datasets. This consolidated table benchmarks the proposed EmoShiftNet against traditional baselines such as FFNN and RNN, as well as state-of-the-art graph-based models like DialogueGCN and DialogueCRN. By evaluating across two diverse datasets, the table highlights the generalizability and effectiveness of EmoShiftNet in capturing emotional nuances across different conversational settings.

**Table 5:** Average F1-score comparison across models on MELD and IEMOCAP datasets.

Model	MELD	IEMOCAP
	Average F1	Average F1
EmoshiftNet	**0.6075**	**0.6885**
DialogueCRN	0.5867	0.5974
DialogueGCN	0.5207	0.6401
FFNN	0.5863	0.6844
RNN	0.5885	0.4351

This table summarizes the weighted average F1-scores achieved by baseline models and EmoShiftNet on two benchmark datasets. Bold font denotes the best performances.

### 4.2 Impact of fusion strategies

**Figure 6:**
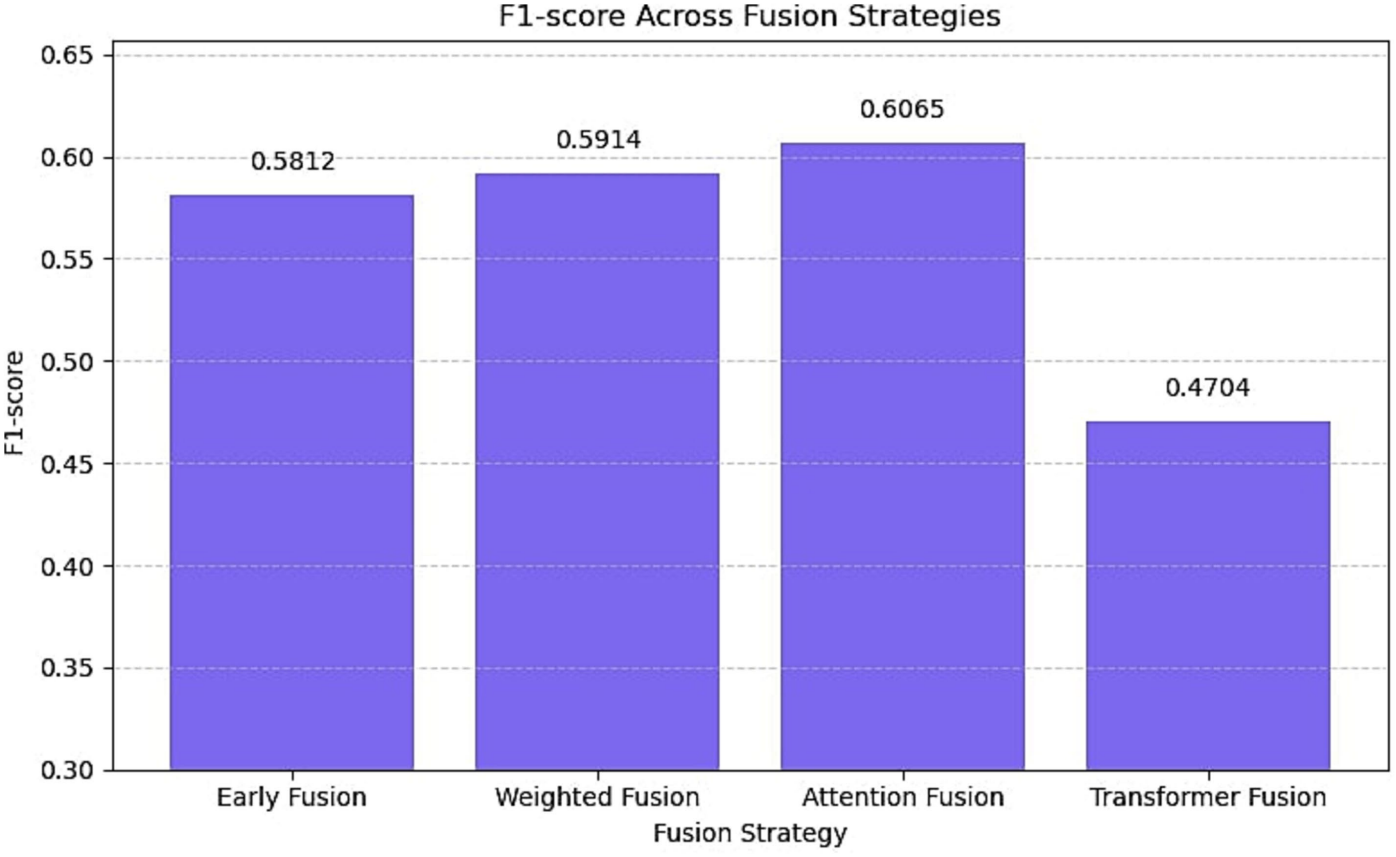
F1-score comparison of EmoShiftNet using different fusion strategies on the filtered MELD dataset. The figure illustrates the performance of four fusion methods—Early Fusion, Weighted Fusion, Attention Fusion, and Transformer Fusion—highlighting that Attention Fusion yields the highest F1-score.

Figure 6 presents the outcomes of experiments conducted with various multimodal fusion strategies applied within the EmoShiftNet framework. The fusion methods evaluated include early fusion, weighted fusion, attention-based fusion, and transformer-based fusion. Each strategy was applied to the same input feature set to ensure fair comparison, and F1-scores are reported.

### 4.3 Class-wise performance analysis

To analyze the performance of different models across individual emotion categories, Table 6 presents a class-wise F1-score comparison for five models: FFNN, RNN, DialogueGCN, DialogueCRN, and the proposed EmoShiftNet. This table provides insights into how each model handles both majority and minority emotion classes on the MELD dataset, highlighting the improvements brought by multimodal fusion and shift-aware learning in EmoShiftNet.

**Table 6:** Class-wise F1-score comparison on MELD dataset.

Model	Anger (153)	Joy (161)	Neutral (469)	Sadness (111)	Surprise (149)
EmoshiftNet	0.38	**0.53**	**0.81**	0.23	**0.61**
EmoShiftNet (without optimization)	0.27	0.49	0.78	0.19	0.55
DialogueCRN	**0.40**	0.51	0.76	0.23	0.49
DialogueGCN	0.31	0.45	0.77	**0.55**	0.42
FFNN	0.36	0.42	0.58	0.28	0.29
RNN	0.40	0.45	0.60	0.32	0.58

The table presents F1-scores for each emotion class across different models evaluated on MELD dataset. Bold font denotes the best performances.

### 4.4 Effect of loss function configurations

To assess the contribution of multi-task loss components, we conducted controlled experiments using different loss setups. Table 7 reports result with variations including focal loss, weighted focal loss, contrastive loss, triplet loss, and the triplet + label smoothing setup.

**Table 7:** Comparison of loss configurations used in training.

Loss strategy	Emotion accuracy	Emotion F1 score
Focal loss	0.5054	0.4203
Weighted focal	0.1053	0.0650
Triplet loss only	0.5863	0.4334
Triplet + Label Smoothing	0.5950	0.4564

Performance metrics are shown for standard cross-entropy, focal loss, weighted focal loss, and the final label-smoothed triplet loss configuration.

### 4.5 Class imbalance mitigation

**Figure 7:**
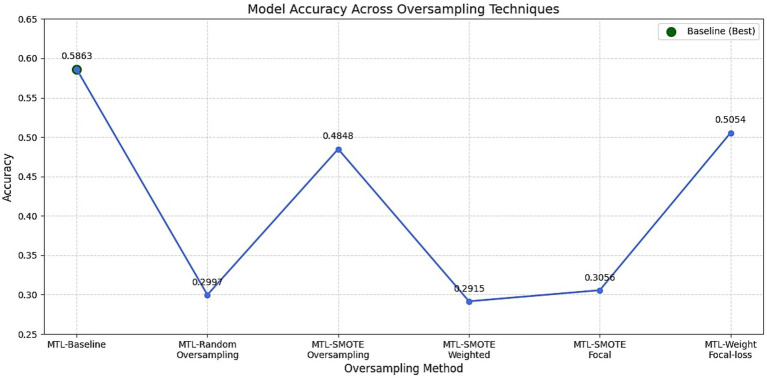
Comparison of oversampling strategies on model performance. The figure presents the impact of random oversampling and SMOTE on emotion classification accuracy.

**Figure 8:**
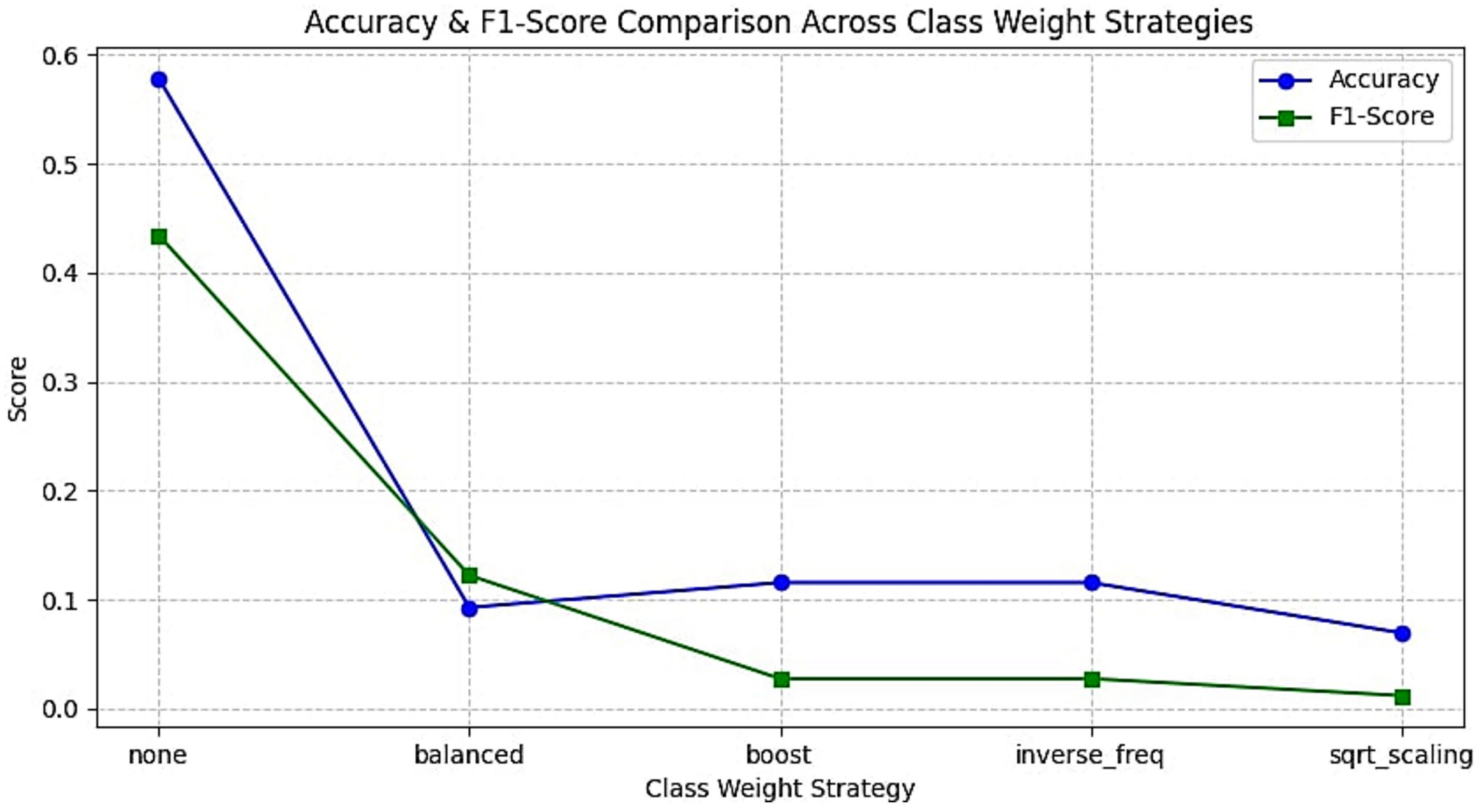
Effect of class weighting methods on model performance. This figure displays the accuracy and F1-scores for various loss reweighting schemes including balanced, boosted, inverse frequency, and square root scaling.

**Figure 9:**
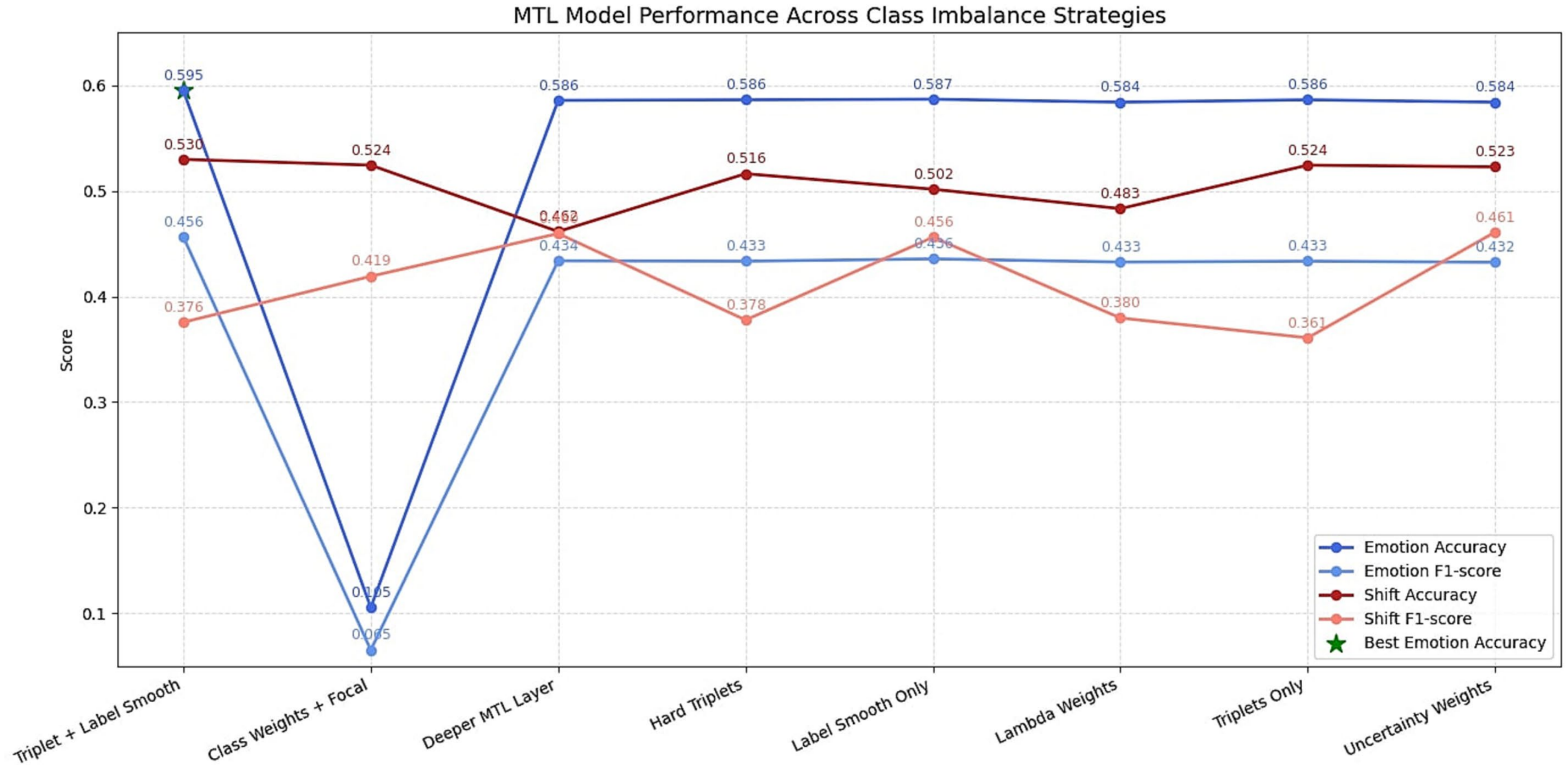
Summary of class imbalance mitigation experiments. The figure provides a consolidated view of different loss function strategies, oversampling methods, and architectural variations used to address label imbalance in MELD.

To mitigate the effects of class imbalance, especially the overrepresentation of the neutral emotion in MELD, several strategies were tested. These included oversampling methods, class weighting schemes, and alternative loss functions. The results of different oversampling methods are shown in Figure 7 and the F1 scores of different class weighting strategies are depicted in Figure 8. In addition to the class weight experiments, several strategies were carried out to mitigate the class imbalance problem of the dataset. A detailed summary of the results obtained on each mitigating mechanism is provided in Figure 9.

### 4.6 Feature contribution and contextual modeling

**Figure 10:**
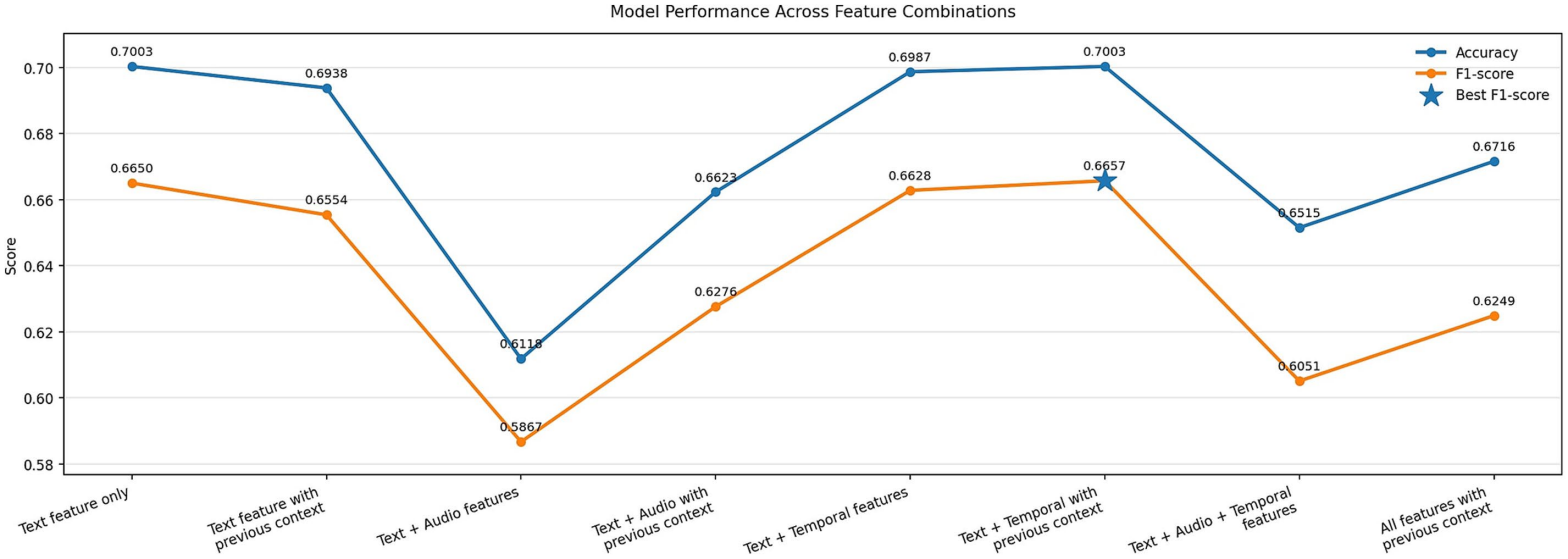
Impact of feature combinations on EmoShiftNet performance. The bar plot compares emotion classification results for different input configurations, including text, audio, temporal features, and context from previous utterances.

Figure 10 presents the performance of EmoShiftNet across different input feature configurations to assess the impact of text, audio, temporal, and contextual signals on emotion recognition and shift detection tasks.

### 4.7 Error analysis

**Figure 11:**
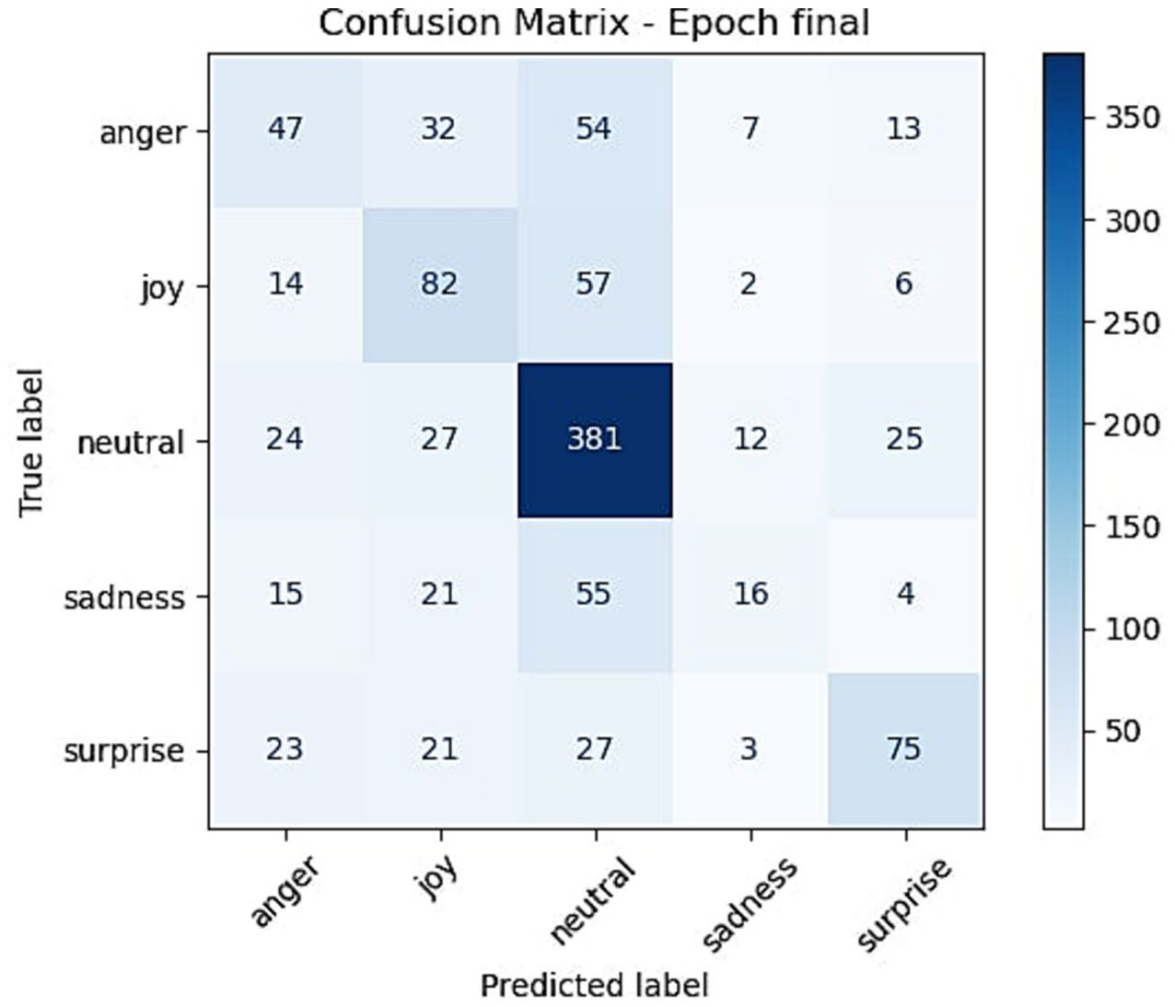
Confusion matrix of EmoShiftNet predictions on the MELD test set. Rows represent true labels, and columns represent predicted labels.

To support deeper evaluation of the proposed framework, we include both quantitative and qualitative error analyses. Figure 11 presents the confusion matrix for EmoShiftNet on the MELD test set, illustrating the distribution of predicted versus actual emotion classes. In addition, Table 8 provides representative examples of misclassified and shift-detection errors observed during evaluation. These samples were selected to help highlight potential challenges in real-world conversation modeling and guide future improvements.

**Table 8:** Sample misclassified utterances from the MELD development set.

Dialogue_ID	Utterance_ID	Text	Predicted emotion	True emotion
16	10	We’re so sorry.	neutral	sadness
16	11	That’s all right, we-we do not need you. In fact, hey I’m over it already.	neutral	anger
19	2	This is all your fault.	sadness	anger
58	4	Hey!	neutral	surprise
63	5	Okay Ross, can I uh, can I ask you something?	neutral	sadness

The table includes five representative cases where the predicted emotion differs from the true label. These examples illustrate typical errors particularly in emotionally subtle or ambiguous expressions.

### 4.8 Emotion shift detection ablation study

**Figure 12:**
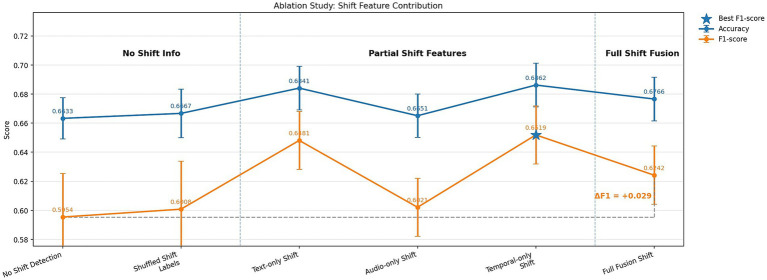
Ablation study on the role of emotion shift detection. The chart compares emotion classification accuracy and F1-score for three variants: full shift-aware model, model without shift detection, and model with randomized shift labels.

To evaluate the contribution of emotion shift detection to overall model performance, an ablation study was conducted using three configurations of EmoShiftNet. These included the full model with shift detection enabled, a variant with the shift detection head removed, and a control version where shift labels were randomly shuffled to remove meaningful supervision. This setup was designed to isolate the specific impact of emotion shift detection in the multi-task learning framework. The outcomes of these experiments are summarized in Table 9 and illustrated in Figure 12, which visualizes the comparative performance across configurations. This ablation highlights the structural role of shift detection in enhancing the shared representation learning within EmoShiftNet.

**Table 9:** Results of the emotion shift detection ablation study.

Configuration	Emotion accuracy	Emotion F1 score
No shift detection	0.6633	0.5954
Shuffled shift labels	0.6667	0.6008
Full shift detection	0.6862	0.6519

The table compares classification accuracy and F1-score across three model variants: without shift detection, with randomized shift labels, and the full shift-aware model.

## 5 Discussion

This section discusses the experimental findings presented in section 4, focusing on performance patterns across emotion shift detection, multimodal fusion, MTL, and feature design.

### 5.1 Effectiveness of shift-aware multi-task learning

To address the challenges of emotion recognition in dynamic, multi-party conversations, EmoShiftNet was benchmarked against four established models—FFNN, RNN, DialogueGCN, and DialogueCRN—on two datasets: MELD and IEMOCAP. All models were evaluated using five consistent emotion classes (neutral, joy, anger, sadness, surprise) to ensure a fair comparison.

As shown in Table 5, EmoShiftNet outperformed all baselines, achieving the highest F1-scores on both datasets (MELD: 0.6075, IEMOCAP: 0.6885). FFNN and RNN performed competitively on MELD (0.5863 and 0.5885), but RNN’s performance dropped on IEMOCAP (0.4351), revealing sensitivity to dialogue complexity. DialogueGCN struggled on MELD (0.5207) but improved on IEMOCAP (0.6401), while DialogueCRN showed stable yet lower scores across both datasets.

These results highlight EmoShiftNet’s robustness and generalization across diverse speaker contexts, enabled by its attention-based fusion and emotion shift modeling. Unlike graph-based or sequential models, it consistently performs well even under label and modality simplification—underscoring the strength of its multi-task design.

Overall, these findings address *RQ1: What are the limitations of current Emotion Recognition in Conversation models in multi-party settings?*—by revealing that traditional ERC models degrade under constrained settings, whereas EmoShiftNet maintains reliability through integrated learning of emotional states and their transitions.

### 5.2 Fusion strategies and modality contribution

To evaluate the impact of different fusion strategies in multimodal emotion recognition, we tested four configurations within the EmoShiftNet framework—early fusion, weighted fusion, attention-based fusion, and transformer-based fusion—while holding all other settings constant. F1-scores on the MELD dataset are reported in Figure 6.

Among them, attention-based fusion performed best (F1 = 0.6065), effectively leveraging contextual cues by dynamically weighing inputs from text, audio, and temporal modalities. Weighted (0.5914) and early fusion (0.5812) offered moderate improvements, highlighting the advantage of context-sensitive integration. Transformer-based fusion underperformed (F1 = 0.4704), likely due to overfitting and inadequate modeling of cross-modal dependencies given the small dataset and shallow architecture. These results affirm that attention-based fusion provides the most stable and effective multimodal integration, particularly when emotional signals are unevenly distributed across modalities.

Used alongside multi-task learning, this fusion method further strengthens contextual emotion modeling. In addressing *RQ3: What techniques are most effective for detecting both subtle and abrupt emotional shifts in conversations?*, attention-based fusion emerges as the most balanced and interpretable technique for detecting both subtle and abrupt emotional shifts in conversation.

### 5.3 Class-wise performance analysis

To examine how models manage class imbalance in emotion recognition, we analyzed class-wise F1-scores for five key emotions—anger, joy, neutral, sadness, and surprise—summarized in Table 6. Comparisons were made between the optimized and unoptimized versions of EmoShiftNet, as well as baseline models including DialogueGCN, DialogueCRN, FFNN, and RNN.

The early version of EmoShiftNet, without enhancements like attention fusion, loss customization, or multi-task learning, underperformed across all classes—particularly for anger (0.27) and sadness (0.19), consistent with known challenges in modeling minority emotions. In contrast, the optimized model significantly improved these scores, with +0.11 gain in anger and +0.04 in sadness, and outperformed DialogueGCN and DialogueCRN in majority classes like neutral (0.81 vs. 0.77, 0.76) and surprise (0.61 vs. 0.52, 0.49).

While DialogueGCN showed decent recall in sadness (0.55), it struggled with anger (0.31). DialogueCRN achieved balanced but weaker scores across the board, indicating its limitations in capturing nuanced emotional cues.

These findings support *RQ2: How does the proposed framework compare with existing ERC models in terms of performance?* by demonstrating that EmoShiftNet not only boosts overall performance but also delivers class-specific improvements, especially for underrepresented emotions. Gains over the unoptimized version further validate the impact of its architectural and loss design. Nonetheless, the persistent gap in low-frequency classes suggests the potential for further improvements through contrastive learning or fine-grained sample reweighting.

### 5.4 Loss strategy and architecture variants

As shown in Figure 9, the optimal results came from adjusting the training objective itself. The final loss configuration—triplet loss combined with label-smoothed cross-entropy—achieved the best balance between accuracy of 0.595 and F1-score of 0.456. This setup allowed the model to learn better inter-class separation in the embedding space while avoiding overconfidence in high-frequency classes. Other strategies like hard triplet mining or deeper MTL layers offered marginal gains or led to instability, confirming the need for carefully designed auxiliary losses. Notably, attempts to apply uncertainty-based loss weighting or adaptive *λ*-weighting did not outperform static configurations, suggesting that stability and simplicity may be more beneficial than dynamic adjustments in low-resource emotional contexts

### 5.5 Class imbalance mitigation strategies

Class imbalance remains a persistent challenge in ERC, particularly in datasets like MELD where the “neutral” class dominates the label distribution as shown in Figure 2. This imbalance skews model training and disproportionately reduces the recall for minority emotions such as fear and disgust. To address this, we experimented with several imbalance mitigation techniques, including oversampling, class weighting, and loss strategy modifications.

#### 5.5.1 Oversampling results

As depicted in Figure 7, the first set of experiments applied random oversampling and SMOTE to artificially increase the frequency of minority emotion classes. However, both approaches led to diminished model performance. Random oversampling drastically reduced emotion accuracy from 0.5863 with the baseline model to 0.2997, due to overfitting on duplicated samples. SMOTE showed moderate improvement with 0.4848 but still underperformed relative to the baseline. More complex strategies like combining SMOTE with focal loss or weighted objectives further decreased performance, indicating poor constructive interaction between synthetic data generation and gradient rebalancing techniques. These results reinforce findings by Buda et al. (2018), which suggest that oversampling can introduce redundant or noisy data that hinders generalization in deep learning models.

#### 5.5.2 Class weighting strategies

Then we explored adjusting the loss function by assigning higher weights to underrepresented classes using various strategies: balanced, inverse frequency, square-root scaling, and boosted weights. As per Figure 8, all weighting strategies degraded performance compared to the unweighted baseline. The “balanced” configuration, intended to equalize class contributions, caused model collapse with an F1-score below 0.1. While inverse frequency and square-root scaling improved minority precision slightly, they severely harmed recall, resulting in suboptimal overall F1-scores. This outcome aligns with the findings of Johnson and Khoshgoftaar (2019), who noted that improperly tuned class weights can destabilize gradient updates and over-penalize rare class misclassifications in MTL setups.

With these experiments, *RQ3: What techniques are most effective for detecting both subtle and abrupt emotional shifts in conversations?* is further addressed. It becomes clear that rather than naive oversampling or reweighting, emotion shift-aware multi-task models benefit more from embedding-level regularization with triplet loss and soft decision boundaries with label smoothing (Cao et al., 2019), which together promote better generalization and minority class recall in noisy conversational settings.

### 5.6 Feature representation analysis

To assess the role of different input modalities, we experimented with various feature combinations—from unimodal (text-only) to multimodal setups incorporating audio, temporal, and contextual cues (see Figure 10). The best results came from the text + temporal + previous utterance setup, which achieved the highest F1-score (0.6657) and matched the classification accuracy of the text-only model (0.7003). This underscores the value of contextualized text features, especially when enriched with dialogue history and temporal patterns like pauses and overlaps (Hazarika et al., 2018b).

In contrast, adding acoustic features (MFCCs, pitch, loudness) led to a performance drop (F1 = 0.5867). This likely stems from their limited capacity to capture high-level emotional content and the noisy nature of MELD’s audio due to overlapping speech and background interference.

Furthermore, full feature fusion failed to improve results, suggesting that context-aware and selective combinations are more effective than exhaustive modality stacking. These findings support *RQ3: What techniques are most effective for detecting both subtle and abrupt emotional shifts in conversations?* by reinforcing the effectiveness of temporal and contextual modeling in detecting emotional shifts.

### 5.7 Error analysis

To better understand EmoShiftNet’s strengths and limitations, we conducted both quantitative and qualitative error analyses. Figure 11 shows the confusion matrix for the MELD development set (1,043 utterances), mapping true versus predicted labels across five emotions: anger, joy, neutral, sadness, and surprise. As expected, the dominant “neutral” class achieved the highest true positives (381), reflecting a model bias toward frequent classes. Conversely, minority classes like sadness and anger were frequently misclassified—anger was often confused with neutral (54 cases) and joy (32), while nearly half of the sadness utterances were misclassified as neutral (55 cases), indicating challenges in detecting nuanced or restrained emotional expressions.

Table 8 provides representative misclassified examples, revealing typical failure cases when emotions are masked or indirectly conveyed. For instance, the sarcastic remark “That’s all right, we-we do not need you. In fact, hey, I’m over it already.” was labeled as neutral instead of anger, likely due to its calm tone. Similarly, empathetic expressions like “We’re so sorry.” were mistaken for neutral rather than sadness, possibly due to ambiguous phrasing or subtle vocal cues.

Overall, this analysis underscores the difficulties in modeling imbalanced and emotionally subtle dialogues. While EmoShiftNet improves overall accuracy, it still struggles with minority classes—addressing *RQ1* and *RQ2* by showing both its advantages and the remaining challenges in nuanced emotion recognition.

### 5.8 Impact of emotion shift detection (ablation study)

The ablation results presented in Table 9 and Figure 12 clearly demonstrate the impact of incorporating emotion shift detection as an auxiliary task. The full EmoShiftNet model with supervised shift labels achieved an accuracy of 0.6766 and an F1-score of 0.6242, marking a notable +0.029 gain in F1 over the no-shift variant (F1 = 0.5954, accuracy = 0.6633). This indicates that modeling emotional transitions contributes meaningfully to contextual understanding, beyond acting as a regularizer. A control experiment using shuffled shift labels yielded an F1 of just 0.6008—comparable to the baseline—confirming that the gains arise from learning coherent emotional flows rather than task augmentation alone.

Among unimodal inputs for shift detection, temporal-only features (e.g., pauses, overlaps) led to the best F1 (0.6519), outperforming both text-only and audio-only variants. Although full multimodal fusion produced stable results, it did not surpass the simpler temporal-only setup, suggesting that the salience of features matters more than raw modality count. These findings reinforce *RQ4: How can incorporating emotion shift detection improve ERC performance?* By explicitly modeling emotional transitions, EmoShiftNet enhances contextual awareness and achieves more accurate recognition in multi-party dialogue.

### 5.9 Ethical considerations

The model was trained using publicly available and ethically sourced data from MELD, based on scripted dialogues from a television show and IEMOCAP, which consists of acted conversational scenarios collected with informed participant consent. No private or sensitive personal data was involved. However, as with all emotion-aware AI systems, responsible deployment is critical to avoid misuse in emotionally sensitive or privacy-critical applications.

### 5.10 Limitations

Despite improvements, class imbalance—particularly the dominance of neutral emotion—remains a challenge. Oversampling and reweighting techniques did not consistently improve performance, emphasizing the need for more advanced imbalance-handling methods. Additionally, acoustic features contributed limited value due to their variability in spontaneous dialogue. The use of handcrafted acoustic features limits the model’s ability to capture nuanced prosodic and paralinguistic cues. Advanced self-supervised audio encoders such as HuBERT (Hsu et al., 2021) and wav2vec (Baevski et al., 2020) have shown promising results in capturing high-level semantic and prosodic information from raw waveforms. Future work could integrate these pretrained models to obtain richer audio representations, potentially improving model performance, especially in noisy conversational contexts

The current framework does not yet incorporate visual features, which could be useful in modeling non-verbal emotional cues. Future work will focus on integrating speaker tracking, richer contextual modeling, and adaptive multimodal fusion techniques to address these gaps.

## 6 Conclusion

This study introduced EmoShiftNet, a shift-aware MTL framework designed to improve emotion recognition in multi-party conversations by jointly modeling emotion classification and emotion shift detection. Through comprehensive experiments on the MELD dataset, the framework demonstrated strong generalization under imbalanced label distributions and complex conversational dynamics. Key findings show that incorporating shift detection as an auxiliary task consistently enhanced context modeling and classification performance. Attention-based fusion proved most effective in integrating textual, temporal, and acoustic features, while the combination of label-smoothed cross-entropy and triplet loss improved discrimination across emotion classes, particularly underrepresented ones. Temporal features and short-term context (e.g., previous utterances) significantly contributed to recognition accuracy, whereas acoustic inputs had limited impact due to variability in spontaneous speech.

Future work can further strengthen EmoShiftNet by addressing current limitations and enhancing its modeling capabilities. This includes exploring advanced imbalance-handling techniques such as curriculum learning and contrastive sampling, integrating speaker-aware mechanisms like graph-based context modeling, and incorporating visual features for richer multimodal fusion. Another promising direction is to expand contextual modeling beyond a single previous utterance by incorporating longer dialogue histories or multiple preceding turns to better capture long-range dependencies and discourse-level emotion shifts. Additionally, dynamic and adaptive fusion strategies may offer better alignment across modalities in real-world conversations. These directions aim to refine the robustness, interpretability, and generalizability of shift-aware ERC systems, contributing toward deeper understanding and more emotionally intelligent dialogue modeling.

## Data Availability

Publicly available datasets were analyzed in this study. This data can be found at: https://affective-meld.github.io/; https://sail.usc.edu/iemocap/iemocap_release.htm.
